# TRPM2 Is Not Required for T-Cell Activation and Differentiation

**DOI:** 10.3389/fimmu.2021.778916

**Published:** 2022-01-14

**Authors:** Niels C. Lory, Mikolaj Nawrocki, Martina Corazza, Joanna Schmid, Valéa Schumacher, Tanja Bedke, Stephan Menzel, Friedrich Koch-Nolte, Andreas H. Guse, Samuel Huber, Hans-Willi Mittrücker

**Affiliations:** ^1^ Department for Immunology, University Medical Center Hamburg-Eppendorf, Hamburg, Germany; ^2^ Hamburg Center for Translational Immunology (HCTI), University Medical Center Hamburg-Eppendorf, Hamburg, Germany; ^3^ Section of Molecular Immunology and Gastroenterology, I. Department of Medicine, University Medical Center Hamburg-Eppendorf, Hamburg, Germany; ^4^ The Calcium Signalling Group, Department of Biochemistry and Molecular Cell Biology, University Medical Center Hamburg-Eppendorf, Hamburg, Germany; ^5^ Mildred Scheel Cancer Career Center HaTriCS4, University Medical Center Hamburg-Eppendorf, Hamburg, Germany

**Keywords:** TRPM2, T cells, T-cell activation, TCR signaling, ADPR, calcium signaling

## Abstract

Antigen recognition by the T-cell receptor induces a cytosolic Ca^2+^ signal that is crucial for T-cell function. The Ca^2+^ channel TRPM2 (transient receptor potential cation channel subfamily M member 2) has been shown to facilitate influx of extracellular Ca^2+^ through the plasma membrane of T cells. Therefore, it was suggested that TRPM2 is involved in T-cell activation and differentiation. However, these results are largely derived from *in vitro* studies using T-cell lines and non-physiologic means of TRPM2 activation. Thus, the relevance of TRPM2-mediated Ca^2+^ signaling in T cells remains unclear. Here, we use TRPM2-deficient mice to investigate the function of TRPM2 in T-cell activation and differentiation. In response to TCR stimulation *in vitro*, *Trpm2* ^-/-^ and WT CD4^+^ and CD8^+^ T cells similarly upregulated the early activation markers NUR77, IRF4, and CD69. We also observed regular proliferation of *Trpm2* ^-/-^ CD8^+^ T cells and unimpaired differentiation of CD4^+^ T cells into Th1, Th17, and Treg cells under specific polarizing conditions. *In vivo*, *Trpm2* ^-/-^ and WT CD8^+^ T cells showed equal specific responses to *Listeria monocytogenes* after infection of WT and *Trpm2* ^-/-^ mice and after transfer of WT and *Trpm2* ^-/-^ CD8^+^ T cells into infected recipients. CD4^+^ T-cell responses were investigated in the model of anti-CD3 mAb-induced intestinal inflammation, which allows analysis of Th1, Th17, Treg, and Tr1-cell differentiation. Here again, we detected similar responses of WT and *Trpm2* ^-/-^ CD4^+^ T cells. In conclusion, our results argue against a major function of TRPM2 in T-cell activation and differentiation.

## Introduction

T-cell receptor (TCR) stimulation causes a rapid increase of the free cytoplasmic Ca^2+^ concentration. The intensity of this Ca^2+^ signal correlates with the strength of the TCR signal and has strong impact on the activation and differentiation processes of T cells (1–4). Triggering of the TCR induces the rapid formation of the 2^nd^ messengers nicotinic acid adenine dinucleotide phosphate (NAADP), cyclic adenosine 5′-diphosphate-ribose (cADPR), and D-*myo*-inositol 1,4,5-trisphosphate (IP_3_). NAADP targets ryanodine receptor 1 (RYR1) in the ER membrane and possibly two pore channels in the lysosomal membranes resulting in the formation of Ca^2+^ microdomains within milliseconds. Subsequent activation of IP_3_ receptors and RYR in the ER membrane by IP_3_ and cADPR further enhances Ca^2+^ release from the ER. Ca^2+^ depletion of the ER is sensed by stromal interaction module-1 (STIM1) which activates entry of extracellular Ca^2+^ *via* ORAI/CRAC channels in the plasma membrane and thereby causes the extended and global cytoplasmic Ca^2+^ signal required for effective T-cell activation (5–8).

Transient receptor potential cation channel subfamily M member 2 (TRPM2) was also identified as Ca^2+^ channel in T cells, but its function in T cells and particularly in TCR signaling is still unclear. TRPM2 is a Ca^2+^-permeable non-selective cation channel in the plasma membrane. The channel is expressed in cells of the central nervous system but is also found in leukocytes, particularly in cells of the myeloid lineage, e.g., neutrophils, macrophages, and dendritic cells (DCs). In these cells, TRPM2 has been linked to diverse functions, such as oxidative stress response, phagosome maturation, and migratory processes (9–15). *Trpm2* mRNA and TRPM2 protein have been detected in T cells (16–19); however, compared to cells of the myeloid lineage, mRNA expression is relatively low in all analyzed T-cell subsets [**Supplementary Figure 1** and www.immgen.org (20)].

TRPM2 is activated by adenosine 5′-diphosphate-ribose (ADPR), and recently, high-resolution structures of TRPM2 with bound ADPR were reported (18, 21–23). Using ADPR microinjection and uncaging of ADPR derivatives, activation of TRPM2 by ADPR was demonstrated in T cells (16–18, 24). Endogenous ADPR is also detected in T cells, and strong stimulation of T cells causes an increase in the ADPR concentration (17). NAADP and cADPR have been described as further agonists for TRPM2, either alone or in synergy with ADPR; however, the activity of these nucleotides on TRPM2 is controversial (5, 16, 25). TRPM2 can also sense reactive oxygen species, although the relevance of this function for T cells is unclear (19, 26). Recently, 2′-deoxyadenosine 5′-diphosphoribose (2dADPR) was identified as a further TRPM2 agonist in T cells. 2dADPR can be isolated from T cells and was more potent than ADPR in stimulation of TRPM2 currents (27).

Currently, hydrolysis of NAD by the NAD-glycohydrolase CD38 is considered to be the main source of ADPR in T cells and CD38 might also be required for 2dADPR formation (11, 25). CD38 is a type II transmembrane protein with its enzymatic activity in the extracellular part. However, a fraction of CD38 is found with an inverted orientation and thus is able to catalyze ADPR formation in the cytoplasm (28). In addition, ADPR may be cleaved from poly- or mono-ADP-ribosylated proteins. In conclusion, these data demonstrate an ADPR/2dADPR–TRPM2 pathway in T cells that can facilitate a Ca^2+^ influx into the cytoplasm (11, 25). However, these results are largely derived from cell lines, e.g., Jurkat cells, and rely on rather artificial activation protocols. Therefore and in light of the relatively low expression of *Trpm2* in primary T cells, it is unclear to which extent they represent a relevant function of TRPM2 in primary T cells.

TRPM2-deficient (*Trpm2* ^-/-^) mice are viable and fertile (29, 30). Interestingly, TRPM2 deficiency profoundly affects the immune system of these mice. *Trpm2* ^-/-^ mice are highly susceptible in several bacterial infection models (14, 30–33). On the other hand, mice show milder disease in inflammation and autoimmune models (19, 29, 34, 35). In most of these studies, altered susceptibility to disease can be linked to an impaired function of granulocytes, macrophages, or dendritic cells. So far, only two studies specifically analyzed T cells from *Trpm2* ^-/-^ mice. Wolf and colleagues found similar early Ca^2+^ responses following TCR stimulation of CD4^+^ T cells from WT and *Trpm2* ^-/-^ mice (7). In contrast, Melzer et al. observed reduced proliferation and cytokine production of total spleen cells and of purified CD4^+^ T cells from *Trpm2* ^-/-^ mice (19). To our knowledge, the function of TRPM2 in T cells has not been specifically addressed in mouse models *in vivo*.

Here, we analyze the response of CD4^+^ and CD8^+^ T cells from *Trpm2* ^-/-^ mice *in vitro* and in infection and inflammation models *in vivo*. We show that *Trpm2* ^-/-^ T cells are not impaired in the expression of early activation markers and in proliferation and differentiation to effector T-cell subsets *in vitro*. *In vivo*, *Trpm2* ^-/-^ CD8^+^ T cells show regular responses in the *Listeria monocytogenes* infection model both after infection of *Trpm2* ^-/-^ mice and after transfer of deficient T cells into infected recipients. Following anti-CD3 mAb-induced intestinal inflammation, *Trpm2* ^-/-^ and WT mice develop a similar disease and equally accumulate defined Th-cell subsets in the intestinal mucosa. A largely regular response of *Trpm2* ^-/-^ CD4^+^ T cells to anti-CD3 mAb treatment is also observed after T cell transfer into WT recipients. In conclusion, our results so far suggest that TRPM2 is not required for CD4^+^ and CD8^+^ T-cell activation and differentiation.

## Methods

### Mice

*Trpm2* ^-/-^ mice (*Trpm2* ^tm1Yamo^) (29), *Rag1* ^-/-^ mice (B6.129S7−*Rag1* ^tm1Mom^/J) (36), OT-1 mice [Tg(TcraTcrb)1100Mjb] (37), and CD90.1 congenic mice (B6.PL-*Thy1^a^*/CyJ) were on the C57BL/6 background. All other mice used in this study were derived from intercrosses of these mouse strains. Mice were housed under specific pathogen-free conditions at the University Medical Center Hamburg-Eppendorf. The housing was done under standard conditions with food and water ad libitum in individually ventilated cages. Mice were monitored on a daily basis. Animal experiments were approved by the local committee for animal experiments of the City of Hamburg (registration numbers: N033/2018, N067/2020). Age- and sex-matched mice were used.

### *Listeria monocytogenes* Infection

Mice were i.p. infected with 10^4^ CFU of a *Listeria monocytogenes* strain recombinant for ovalbumin (LmOVA) (38). Inocula were controlled by plating on TSB agar plates. From day 2 on, mice were treated with 2 mg/ml ampicillin in the drinking water. Endogenous T-cell responses were analyzed on day 8 postinfection. For T-cell co-transfer studies, CD90.1 congenic mice or *Rag1* ^-/-^ mice were infected with LmOVA. On the same day, infected mice received a mix of WT and *Trpm2* ^-/-^ OT-1 CD8^+^ T cells. Spleen cells from WT CD90.1^+^CD90.2^+^ OT-1 cells and *Trpm2* ^-/-^ CD90.1^-^CD90.2^+^ OT-1 cells were purified and mixed to reach a 1:1 ratio of CD8^+^ T cells. Recipient mice intravenously received a total of approx. 10,000 CD8^+^ T cells. Responses in CD90.1 congenic and *Rag1* ^-/-^ recipients were analyzed after 5 days and after 8 weeks, respectively. For analysis of endogenous T-cell response and for 8-week transfer experiments, 2 µg per mouse anti-CD45 mAb (30-F11, AF700) mAb was injected i.v., 3 min before sacrificing to label intravascular cells.

### Isolation and Stimulation of Cells

Cells from spleen were isolated by pressing the organ successive through 70- and 40-µm cell strainers. Cells from the kidney, lung, and liver were digested for 40 min at 37°C with 10 U/ml DNase I (Sigma-Aldrich, St. Louis, MO) and 400 µg/ml Collagenase D (Roche, Mannheim, Germany). Leukocytes were enriched by density gradient centrifugation (37.5% Easycoll, Merck Millipore, Darmstadt, Germany) and then filtered through a 30-µm strainer. Erythrocytes were depleted with lysis buffer (155 mM NH_4_Cl, 10 mM KHCO_3_, 10 µM EDTA, pH 7.2). After removal of Peyer’s patches, the small intestine was opened longitudinally and washed in PBS 1% FCS. Then, the small intestine was cut into small pieces of approx. 0.5-cm length and incubated in the presence of 5 mM EDTA in complete medium at 37°C for 30 min while shaking. Intraepithelial lymphocytes (IEL) in the supernatant were collected and enriched by centrifugation. For isolation of lamina propria lymphocytes (LPL), the remaining tissue was digested with collagenase IV (100 U/ml, Roche Diagnostics GmbH, Mannheim) and DNase I (10 U/ml Sigma-Aldrich) in complete medium at 37°C for 45 min while shaking. The digested intestinal tissue was further homogenized by passing through a metal strainer, and pooled IEL and LPL fractions were enriched by a 40%/67% Percoll gradient centrifugation. Lymphocytes were collected from the interphase.

For induction of cytokines, cells were incubated for 4 h in IMDM medium supplemented with fetal calf serum, glutamine, gentamicin, and 2-mercaptoethanol. Cells were simulated for 4 h, with phorbol 12-myristate 13-acetate (PMA, 50 ng/ml, Sigma-Aldrich) and ionomycin (1 µM, Sigma Aldrich) or with the ovalbumin_257-264_ peptide (10^-6^ M, SIINFEKL) (JPT, Berlin, Germany). Brefeldin A (10 µg/ml, Sigma Aldrich) was added to the cultures to prevent cytokine secretion. In controls, medium only contained brefeldin A.

For induction of CD69, IRF4, and NUR77, spleen cells were cultured for 4, 24, or 48 h in 96W plates coated with anti-CD3ϵ mAb (2 µg/ml, clone: 145-2C11). Anti-CD28 mAb (1 µg/ml, clone: 37.51) was added to the culture. In some of the assays, cells were CFSE labeled prior to stimulation. A division index was calculated with the FlowJo software (Tree Star, Ashland, OR, USA). For long-term culture, cells were stimulated with anti-CD3 mAb, antiCD28, mAb and IL-2 (100 U/ml IL-2, Proleukin S, Novartis, Nürnberg, Germany). After 3 days, cells were washed and further cultured with IL-7 (10 ng/ml mIL-7, PeproTech, Hamburg, Germany).

### *In Vitro* Differentiation of CD4^+^ T Cells

Lymphocytes were isolated from spleen and lymph nodes of WT and *Trpm2* ^-/-^ mice. Naive CD4^+^ CD25^-^ CD44^-^ T cells were enriched by depletion of CD25^+^ and CD44^+^ cells followed by enrichment of CD4^+^ T cells using MACS according to the manufacturer’s instruction (Miltenyi Biotec, Bergisch-Gladbach, Germany). The purity of CD4^+^ T cells obtained was about 80% as determined by flow cytometry. For each differentiation condition, the cells were cultured in a 96-well plate at 2 × 10^5^ cells per well in 200 µl of full Click’s medium (Irvine Scientific, Santa Ana, USA) supplemented with cytokines and antibodies. For differentiation of Th1 cells, naive CD4^+^ T cells were cultured in the presence of 100 U/ml mIL-2, 10 ng/ml mIL-12, 10 µg/ml anti-IL-4 mAb (clone: 11B11), and 2 µg/ml anti-CD28 mAb (clone: 37.51) in plates coated with 10 µg/ml anti-CD3ϵ mAb (clone: 145 2C1). For the differentiation of Th17 cells, naive CD4^+^ T cells were cultured in the presence of 10 ng/ml mIL-6 and 0.25 ng/ml hTGF-β1, 10 µg/ml anti-IL-4 mAb, 10 µg/ml anti-IFN−γ mAb (clone: XMG1.2), and 2 µg/ml anti-CD28 mAb in plates coated with 10 µg/ml anti-CD3ϵ mAb. For differentiation of Treg cells, naive CD4^+^ T cells were cultured in the presence of 50 U/ml mIL-2 and 2 ng/ml hTGF-β1 and 2 µg/ml anti-CD28 mAb in plates coated with 2 µg/ml anti-CD3ϵ mAb. For differentiation of Tr1 cells, naive CD4^+^ T cells were cultured in the presence of 30 ng/ml mIL-27 and 0.25 ng/ml hTGF-β1 and 2 µg/ml anti-CD28 mAb in plates coated with 10 µg/ml anti-CD3 mAb. Cytokines and antibodies were purchased from BioLegend (San Diego, CA) and Miltenyi Biotec. After 3 days, T cells were restimulated with PMA (50 ng/ml, Sigma-Aldrich) and ionomycin (1 µM, Sigma-Aldrich) for 4 h in the presence of monensin (2 µM, BioLegend) and expression of cytokines and Foxp3 was determined by intracellular antibody staining.

### Antibody Staining and Flow Cytometry

After isolation from tissue or after cell culture, cells were incubated in PBS with 1% rat serum and 10 µg/ml anti-Fc-receptor mAb (clone 2.4G2, Bio X Cell, West Lebanon, NH). For extracellular staining, fluorochrome-conjugated antibodies and a fixable dead cell stain (AF750 life/dead staining or Pacific Orange succinimidyl ester, Life Technologies, Carlsbad, CA) were added. Cells were incubated for 15 min on ice. Intracellular antibody staining was conducted with the Foxp3/Transcription Factor Staining Buffer Set (eBioscience, Carlsbad, CA) according to the manufacturer’s protocol. Cells were washed with PBS 1% FCS and incubated with antibodies for intracellular staining in PBS 1% FCS for 20 min at RT.

Fluorochrome-conjugated antibodies against murine CD3 (clone 17A2, BV421), CD4 (clone RM4-5, FITC/AF700/BV605/BV785), CD8α (clone 53-6.7, PerCP/BV650), CD38 (clone 90, PE-Cy7), CD40L (clone MR1, PerCP-Cy5.5), CD44 (clone IM7, APC/BV785), CD45 (clone 30-F11, APC-Cy7/AF700/BV510/BV785), CD62L (clone MEL-14, APC/PerCP), CD69 (clone H1.2F3, PE-Cy7/V450/BV785), CD90.1 (clone HIS51, FITC/eFlour 450), CD90.2 (clone 53-2.1, PerCP), CD127 (clone A7R34, BV421), CX3CR1 (clone SA011F11, PE), Ly6C (clone AL-21, FITC), Ly6G (clone 1A8, PerCP-Cy5.5), CTLA4 (clone UC10-4F10-11, PE), PD1 (clone 29F.1A12, BV421), LAG3 (clone C9B7W, APC), IFN-γ (clone XMG1.2, APC-Cy7/BV785), IL-10 (clone JES5-16E3/APC/PE), PE), IL-17A (clone TC11-18H10.1, BV785/AF488/APC), TNF-α (clone MP6-XT22; V450), Foxp3 (clones NRRF-30, PE and FJK-16s, APC), IRF4 (clone 3E4, PE-Cy7), KI-67 (clone SolA15, PE), KLRG1 (clone 2F1, BV605), and NUR77 (clone 12.14, PE) were obtained from BioLegend, eBioscience, Thermo Fisher (Darmstadt, Germany) or BD Bioscience (Heidelberg, Germany). The anti-mouse TRPM2 mAb (clone A128) was developed by DNA immunization of rats with a plasmid expressing the full-length cDNA of mouse TRPM2. For analysis of TRPM2 expression, spleen cells were stained intracellularly with unconjugated anti-TRPM2 mAb and then stained with anti-rat-IgG antibodies (R-PE, polyclonal, Dianova, Hamburg, Germany). APC-conjugated H-2K^b^/SIINFEKL dextramers were obtained from Immudex (Copenhagen, Denmark).

Cells were analyzed using Canto II, Celesta or Fortessa flow cytometers (BD Biosciences, Franklin Lakes, NJ, USA) and FlowJo software (Tree Star, Ashland, OR, USA).

### Anti-CD3 mAb Induced Intestinal Inflammation

Mice were i.p. injected with 15 µg anti-CD3 mAb (clone: 145 2C11) on days 0, 2, and 4. Mice were analyzed 4 h after the last injection. For the T-cell transfer approach, cells were isolated from spleen and peripheral lymph nodes of WT and *Trpm2* ^-/-^ mice. Total CD4^+^ cells were enriched with MACS according to the manufacturer’s instructions. Recipient *Rag1* ^-/-^ mice received 2 × 10^6^ CD4^+^ cells i.v. After 4 weeks, recipients were treated i.p. with 2 µg anti-CD3 mAb on days 0, 2, and 4 and were analyzed 4 h after the last injection.

### Quantitative RT-PCR

CD4^+^ and CD8^+^ T cells were isolated from spleens of WT and *Trpm2* ^-/-^ mice and purified by MACS (Miltenyi Biotec, Bergisch Gladbach, Germany). Total RNA from pooled CD4^+^ and CD8^+^ T cells was isolated using TRIzol^®^ reagent (Invitrogen) according to the manufacturer’s protocol. RNA was reverse transcribed with the high-capacity cDNA synthesis Kit (Thermo Fisher, Darmstadt, Germany). cDNA concentrations for *Ryr1*, *Tpcn1*, *Tpcn2*, *Orai1*, and the control *Hprt* were determined with TaqMan PCR using primer and probes from Thermo Fisher: *Hprt* Mm03024075_m1, *Ryr1* Mm01175211_m1, *Tpcn1* Mm00455326_m1, *Tpcn2* Mm00628260_m1, and *Orai1* Mm00774349_m1.

### Statistics

Statistical analyses were performed with Prism software (GraphPad Software Inc., La Jolla, CA). Results were analyzed with the tests indicated in the figure legends. A p-value of <0.05 was considered significant (p < 0.05) and is indicated with *.

## Results

### TRPM2 Deficiency Does Not Impair CD8^+^ T-Cell Activation *In Vitro*

**Figure 1:**
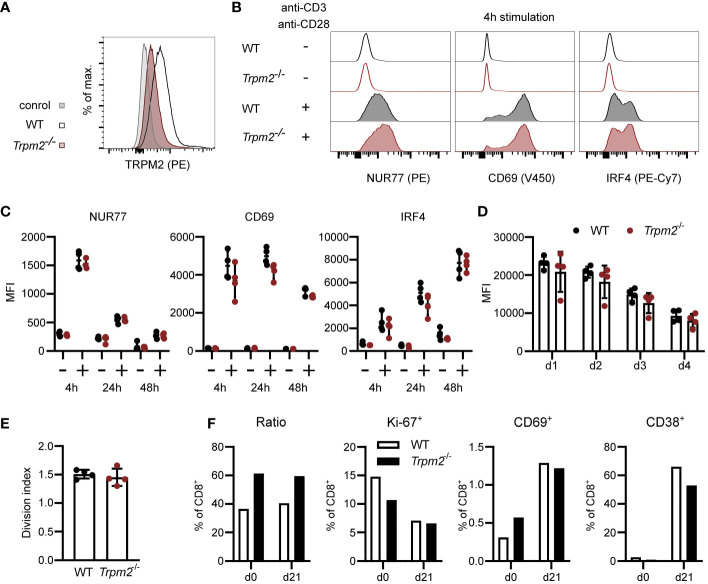
TRPM2 deficiency does not impair CD8^+^ T-cell activation *in vitro*. **(A)** CD8^+^ T cells were intracellularly stained with anti-TRPM2 mAb and PE-conjugated anti-rat IgG antibody (control: WT cells stained only with the secondary antibody). **(B, C)** Spleen cells from WT and *Trpm2* ^-/-^ mice were stimulated for 4, 24, or 48 h with plate-coated anti-CD3ϵ mAb and soluble anti-CD28 mAb. Expressions of CD69, NUR77, and IRF4 were determined by extra- and intracellular antibody staining. Representative staining **(B)** and results **(C)** for CD8^+^ T cells are shown. **(D, E)** Spleen cells were CFSE-labeled and stimulated with anti-CD3ϵ mAb and anti-CD28 mAb. Representative results for the mean fluorescence intensity (MFI) of CFSE of CD8^+^ T cells on days 1–4 **(D)** and the division index for day 4 **(E)** are given. **(F)** WT (CD90.1^+^) and *Trpm2* ^-/-^ (CD90.2^+^) spleen cells were mixed and stimulated with anti-CD3ϵ and anti-CD28 for 3 days. Then, cells were washed and incubated with IL-7. On day 21, cells were analyzed by extra- and intracellular antibody staining. Representative results for the ratio of CD90.1^+^ and CD90.2^+^ CD8^+^ T cells and for the expression of the proliferation markers Ki-67 and of CD69 and CD38 are shown. Bars and scatters in **(C–E)** give the mean ± SEM and were analyzed with the unpaired t-test. All experiments were conducted at least twice.

Ca^2+^ signaling is central for T-cell activation and differentiation, and Ca^2+^ influx facilitated by TRPM2 could enhance or modulate these processes. In a first set of experiments, the expression of TRPM2 was determined in CD8^+^ T cells (**Figure 1A** and **Supplementary Figure 2A**). Compared to Ly6C^+^ and Ly6G^+^ myeloid cells (inflammatory monocytes and neutrophils), we detected only a low level of TRPM2 expression in CD8^+^ T cells from WT mice which was markedly reduced in CD8^+^ T cells from *Trpm2* ^-/-^ mice. Spleen cells from WT and *Trpm2* ^-/-^ mice were stimulated with anti-CD3ϵ and anti-CD28 mAb, and the expression of CD69 and of the transcription factors IRF4 and NUR77 (NR4A1) was determined by flow cytometry. Expression of these proteins is induced within a few hours of TCR stimulation and can be used to determine the quality of the TCR signal. After 4 h, we observed strong upregulation of all three proteins and expression of IRF4 was further increased after 24 and 48 h of culture (**Figures 1B, C**). CD8^+^ T cells from WT and *Trpm2* ^-/-^ mice showed similar upregulation of the proteins. Then, spleen cells were labeled with CFSE and proliferation was determined by loss of the CFSE label. Cells were stimulated with anti-CD3ϵ and anti-CD28 mAb, and after 1, 2, 3, and 4 days, CFSE staining was determined (**Figures 1D, E** and **Supplementary Figure 2B**). T-cell activation resulted in cumulative loss of CFSE staining in CD8^+^ T cells; however, there was no difference in proliferation between WT and *Trpm2* ^-/-^ cells. TRPM2 could also modulate the T-cell response at later time points. Therefore, CD8^+^ T cells from WT and *Trpm2* ^-/-^ mice were mixed and activated with anti-CD3ϵ and anti-CD28 mAb. After 3 days, activated cells were cultured in medium containing IL-7 (**Figure 1F**). After 21 days, CD8^+^ T cells expressed only low levels of CD69 and of the proliferation marker Ki-67 but had upregulated CD38. Importantly, the ratio of WT to *Trpm2^-/-^* CD8^+^ T cells was similar to the ratio at the start of the culture, indicating that the lack of TRPM2 did not alter the T-cell response.

### TRPM2 Deficiency Does Not Impair CD4^+^ T-Cell Activation and Differentiation *In Vitro*

**Figure 2:**
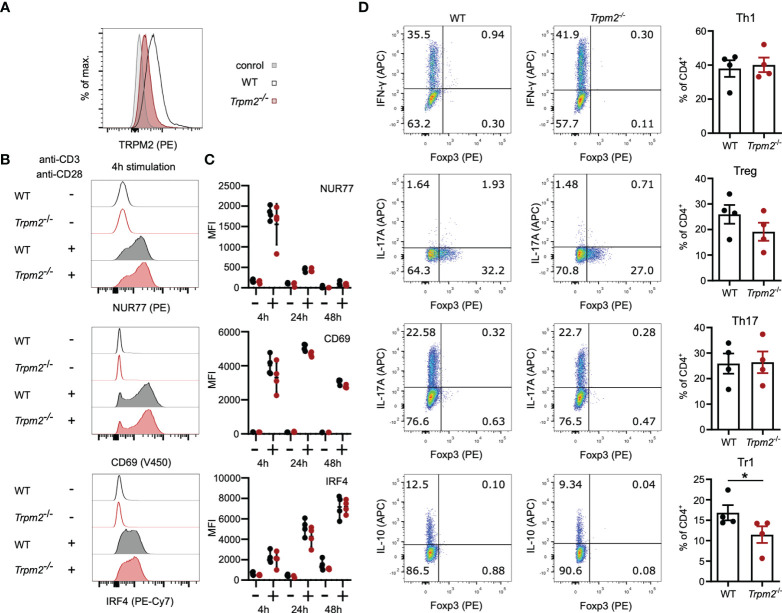
TRPM2 deficiency does not impair CD4^+^ T-cell activation and differentiation *in vitro*. **(A)** CD4^+^ T cells were intracellularly stained with anti-TRPM2 mAb and PE-conjugated anti-rat IgG antibody (control: WT cells stained only with the secondary antibody). **(B, C)** Spleen cells from WT and *Trpm2* ^-/-^ mice were stimulated for 4, 24, or 48 h with plate-coated anti-CD3ϵ mAb and soluble anti-CD28 mAb. Expressions of CD69, NUR77, and IRF4 were determined by extra- and intracellular antibody staining. Representative staining **(B)** and results **(C)** for CD4^+^ T cells are shown. **(C)** Purified naïve CD4^+^ T cells were stimulated with plate-bound anti-CD3ϵ mAb and soluble anti-CD28 mAb and cultured under Th1-, Th17-, Treg-, and Tr1-polarizing conditions. After 4 days, T cells were stimulated with PMA/ionomycin in the presence of monensin, and cytokine and Foxp3 expression was measured by flow cytometry. Shown are representative dot plots and corresponding bar graphs. Th1 cells were defined as IFN-γ^+^ CD4^+^ T cells, Th17 cells as IL-17A^+^ CD4^+^ T cells, Treg cells as Foxp3^+^ CD4^+^ T cells, and Tr1 cells as Foxp3^-^ IL-10^+^ CD4^+^ T cells. Bars and scatters in **(C, D)** give the mean ± SEM and were analyzed with the unpaired t-test. *p < 0.05. All experiments were conducted at least twice.

Similar to WT CD8^+^ T cells, WT CD4^+^ T cells expressed only low levels of TRPM2 and staining was reduced in *Trpm2* ^-/-^ CD4^+^ T cells (**Figure 2A**). The early response following TCR stimulation was also determined in CD4^+^ T cells (**Figures 2B, C**). CD4^+^ T cells showed rapid upregulation of CD69, NUR77, and IRF4, but there was no difference between WT and *Trpm2* ^-/-^ CD4^+^ T cells. We also determined the induction of the inhibitory receptors CTLA4, PD1, and LAG3 (**Supplementary Figure 3**). We observed an equal expression of these receptors on WT and *Trpm2* ^-/-^ CD4^+^ T cells. Differentiation of CD4^+^ Th cells is regulated by cytokine signals from the environment but also by the strength of the TCR stimulus and the quality of the TCR-induced Ca^2+^ signal. Thus, modulation of the Ca^2+^ signal by TRPM2 could influence the fate of Th-cell differentiation. Purified CD4^+^ T cells from WT and *Trpm2* ^-/-^ mice were stimulated under defined conditions to induce IFN-γ^+^ Th1 cells, IL-17A^+^ Th17 cells, Foxp3^+^ regulatory T cells (Treg cells), and Foxp3^-^ IL-10^+^ regulatory 1 T cells (Tr1 cells). After 3 days, T cells were activated with PMA and ionomycin, and expression of Foxp3 and cytokines was determined by intracellular staining and flow cytometry (**Figure 2D**). Consistent with the conditions of differentiation, we detected upregulation of IFN-γ, IL-17A, IL-10, and Foxp3; however, WT and *Trpm2^-/-^* CD4^+^ T cells did not significantly differ in the generation of IFN-γ^+^ Th1 cells, IL-17A^+^ Th17 cells, and Foxp3^+^ Treg cells. In the experiment shown, there was a small reduction of *Trpm2* ^-/-^ Tr1 cells; however, this reduction was not consistent in other experiments.

Absence of TRPM2 could be compensated by higher expression of other Ca^2+^ channels. Therefore, mRNA was isolated from WT and *Trpm2* ^-/-^ T cells and the expression of *Ryr1*, *Tpcn1*, *Tpcn2*, and *Orai1* coding for Ca^2+^ channels that might compensate for the absence in T cells was measured by RT-PCR (**Supplementary Figure 4**). We detected similar mRNA levels for all analyzed Ca^2+^ channels in WT and *Trpm2* ^-/-^ T cells.

In conclusion, our data so far provide no evidence for a substantial role of TRPM2 in the activation and differentiation of CD4^+^ and CD8^+^ T cells.

### TRPM2 Deficiency Does Not Impair CD8^+^ T-Cell Activation *In Vivo*

**Figure 3:**
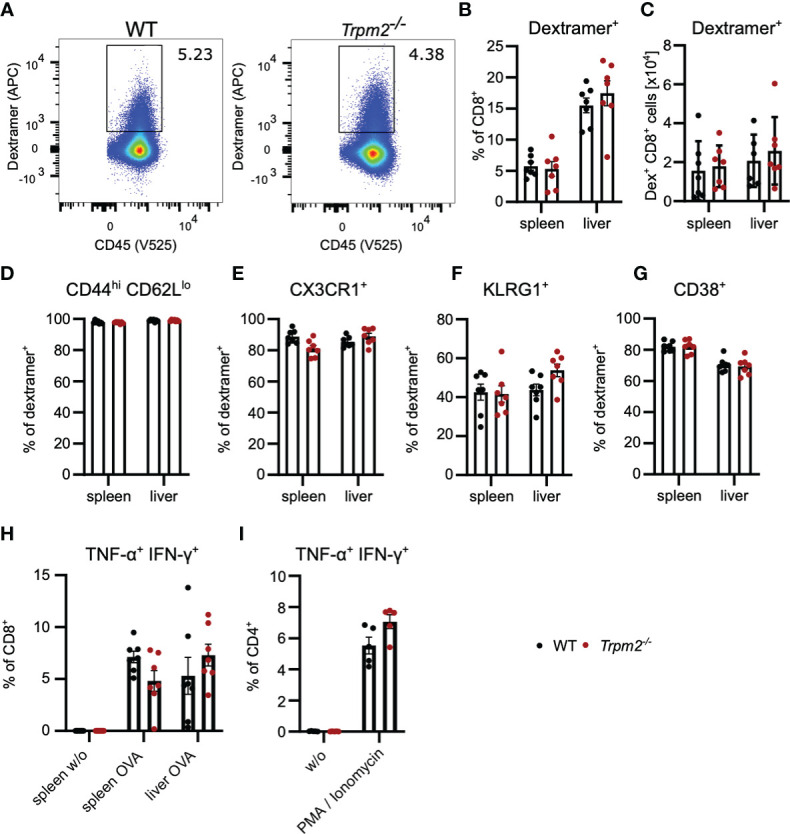
TRPM2 deficiency does not impair the CD8^+^ T-cell response to *L. monocytogenes. Trpm2* ^-/-^ and WT mice were infected with LmOVA. Mice were treated with ampicillin in the drinking water from day 2 on until analysis at day 8. Ovalbumin-specific CD8^+^ T cells were detected with H−2K^b^ OVA_257-264_-dextramers. **(A)** Representative dextramers staining of CD8-gated T cells. **(B)** Frequencies and **(C)** numbers of dextramers^+^ CD8^+^ T cells in spleen and liver of infected mice. **(D–G)** Percentage of CD44^hi^CD62L^lo^ **(D)**, CX3CR1^+^ **(E)**, KLRG1^+^ **(F)**, and CD38^+^ **(G)** cells among dextramer^+^ CD8^+^ T cells. **(H)** Spleen and liver cells were stimulated for 4 h with OVA_257-264_ peptide and the expression of IFN-γ and TNF-α in CD8^+^ T cells was determined by intracellular cytokine staining. **(I)** Spleen cells were stimulated for 4 h with PMA/ionomycin, and the expression of IFN-γ and TNF-α in CD4^+^ T cells was determined by intracellular cytokine staining. Bars in **(C–I)** give the mean ± SEM and were analyzed with the unpaired t-test. Data from one of three experiments are shown.

In order to analyze the role TRPM2 in CD8^+^ T cells *in vivo*, we used the *Listeria monocytogenes* infection model. WT and *Trpm2* ^-/-^ mice were infected with an ovalbumin-recombinant strain of *L. monocytogenes* (LmOVA) which induce a strong CD8^+^ T cell response against the OVA_257-264_ peptide (38). Since *Trpm2* ^-/-^ mice are more susceptible to *L. monocytogenes* (30, 31), mice were treated after 2 days with ampicillin in the drinking water which results in the rapid elimination of Listeria but does only marginally effect the T-cell response (39). Eight days postinfection, frequencies and total numbers of ovalbumin-specific CD8^+^ T cells were determined using OVA_257-264_H-2K^b^ dextramers (**Figures 3A–C**). WT and *Trpm2* ^-/-^ mice showed similar numbers of dextramers^+^ CD8^+^ T cells in spleen and liver, the main sites of listeria replication. Dextramer^+^ CD8^+^ T cells in both mouse strains were CD44^hi^CD62L^lo^ and similar frequencies expressed CX3CR1 and KLRG1, indicative for highly activated effector T cells (**Figures 3D–F**). Dextramer^+^ CD8^+^ T cells did also not differ with regard to the upregulation of CD38 (**Figure 3G**). Spleen and liver cells were also incubated with OVA_257-264_ peptide, and the induction of TNF-α and IFN-γ was determined by intracellular cytokine staining (**Figure 3H**). Again, we observed similar frequencies of TNF-α^+^IFN-γ^+^ CD8^+^ T cells. There was also no difference in the production of IFN-γ and TNF-α by CD4^+^ T cells in response to polyclonal restimulation (**Figure 3I**).

**Figure 4:**
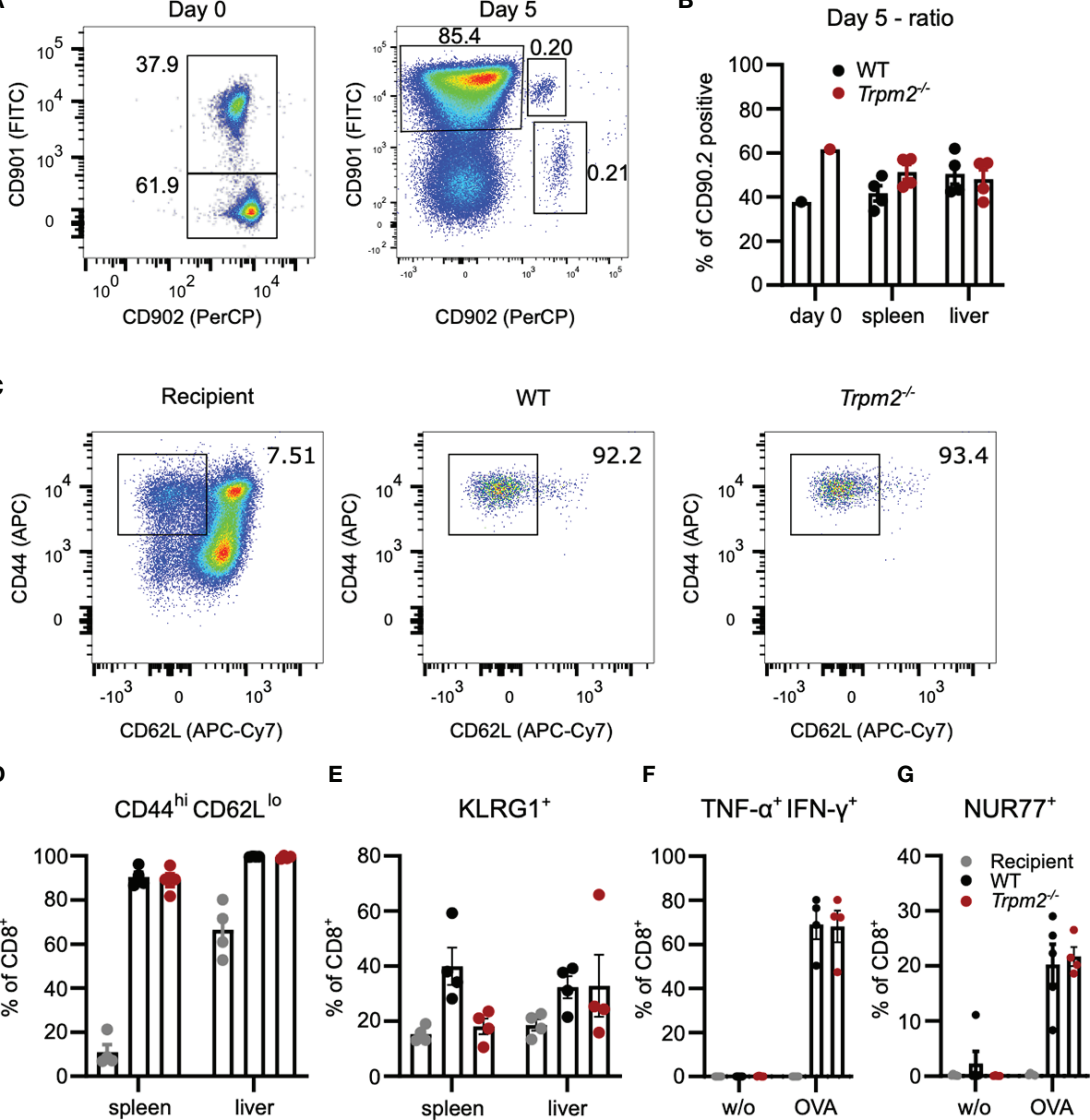
TRPM2 deficiency does not impair the acute CD8^+^ T-cell response to *L. monocytogenes* in a T-cell transfer model. *Trpm2* ^-/-^ CD8^+^ OT-1 T cells (CD90.1^-^CD90.2^+^) and WT CD8^+^ OT-1 T cells (CD90.1^+^CD90.2^+^) were mixed roughly at a ratio 1:1 and 10^4^ CD8^+^ T cells were i.v. transferred to CD90.1^+^CD90.2^-^ WT mice. Recipients were infected on the same day with LmOVA and analyzed 5 days later. **(A)** Representative dot plots for CD90.1 and CD90.2 staining of the transferred CD8^+^ T-cell population (Day 0) and of the CD8^+^ T cells from the spleen at day 7. (In the experiment shown, we transferred cells with a 2:3 ratio of WT to *Trpm2* ^-/-^ CD8^+^ T cells.) **(B)** Frequencies of WT and *Trpm2* ^-/-^ cells among CD8^+^ donor T cells at day 0 (mix before transfer) and day 5 postinfection. **(C)** Representative dot plots for CD44 and CD62L expression of donor and recipient CD8^+^ T cells. **(D)** Frequencies of CD44^hi^CD62L^lo^ donor and recipient CD8^+^ T cells in spleen and liver. **(E)** Frequencies of KLRG1^+^ donor and recipient CD8^+^ T cells in spleen and liver. **(F)** Spleen cells were stimulated for 4 h with OVA_257-264_ peptide and the frequencies of IFN-γ^+^ TNF-α^+^ donor and recipient CD8^+^ T cells was determined by intracellular cytokine staining. **(G)** Frequencies of NUR77^+^ donor and recipient CD8^+^ T cells after OVA_257-264_ peptide stimulation of spleen cells. Bars give the mean ± SEM. Results in **(D–G)** and were analyzed with ANOVA with Dunnett’s multiple-comparison test. Experiments were conducted twice.

More excessive initial inflammation and altered function of TRPM2-deficient innate immune cells could mask a defect of CD8^+^ T cells in *Trpm2* ^-/-^ mice. Therefore, we used a competitive T-cell transfer assay to characterize the function of TRPM2 in CD8^+^ T cells. *Trpm2* ^-/-^ mice were crossed with OT-1 mice which are transgenic for an MHC class I-restricted OVA_257-264_-specific TCR (37). CD8^+^ T cells from WT and *Trpm2* ^-/-^ mice were mixed roughly at a 1:1 ratio, and 1 × 10^4^ CD8^+^ T cells were transferred into recipient mice infected with LmOVA. Donor and recipient cells differed in the expression of CD90.1 and CD90.2, which allowed identification of the different cell populations. Five days post transfer and infection, CD8^+^ T cells derived from both donors could be detected in spleen and liver (**Figures 4A, B**). However, the ratio of WT to *Trpm2* ^-/-^ cells in both tissues was similar to that of the transferred CD8^+^ T-cells and both populations were similar in their expression profiles of CD44, CD62L, and KLRG1 (**Figures 4C–E**). In addition, after stimulation with the OVA_257-264_ peptide, WT and *Trpm2* ^-/-^ OT-1 T-cells showed similar induction of IFN-γ and TNF-α and of NURF77 (**Figures 4F, G**). Thus, TRPM2 deficiency restricted to CD8^+^ T cells did not significantly impair their response during acute infection.

**Figure 5:**
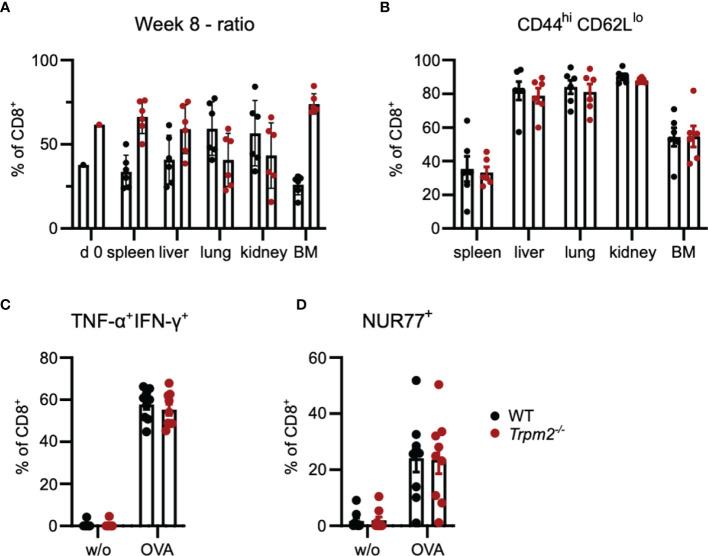
TRPM2 deficiency does not impair the long-term CD8^+^ T-cell response to *L. monocytogenes* in a T-cell transfer model. 5,000 *Trpm2* ^-/-^ CD8^+^ OT-1 T cells (CD90.1^-^CD90.2^+^) and 5,000 WT CD8^+^ OT-1 T cells (CD90.1^+^CD90.2^+^) were mixed and i.v. transferred to *Rag1* ^-/-^ mice. Recipients were infected on the same day with LmOVA and analyzed 8 weeks later. **(A)** Frequencies of WT and *Trpm2* ^-/-^ cells among CD8^+^ donor T cells at day 0 (mix before transfer) and 8 weeks postinfection in spleen, liver, lung, kidney, and bone marrow (BM). **(B)** Frequencies of CD44^hi^CD62L^lo^ donor CD8^+^ T cells in different tissues. **(C)** Spleen cells were stimulated for 4 h with OVA_257-264_ peptide, and the frequencies of IFN-γ^+^ TNF-α^+^ donor and recipient CD8^+^ T cells were determined by intracellular cytokine staining. **(D)** Frequencies of NUR77^+^ donor and recipient CD8^+^ T cells after OVA_257-264_ peptide stimulation of spleen cells. Bars in give the mean ± SEM. Results in **(B–D)** and were analyzed with the unpaired t-test. One representative experiment of three is shown.

The competitive transfer assay was also used to determine the role of TRPM2 in CD8^+^ memory T-cell formation. To exclude rejection of donor cells, *Rag1* ^-/-^ mice were used as recipients. Eight weeks post transfer and LmOVA infection, donor cells in the spleen, liver, lung, kidney, and bone marrow were analyzed. In the spleen, liver, and bone marrow, we observed ratios of WT to *Trpm2* ^-/-^ cells similar to the ratio of the transferred cell population (**Figure 5A**). Interestingly, WT cells were slightly more prominent in lung and kidney, indicating a disadvantage of *Trpm2* ^-/-^ CD8^+^ T cells in migration into or survival within these tissues. Phenotypical characterization revealed similar expression profiles for CD44 and CD62L cells with high frequencies of CD44^hi^CD62L^lo^ effector/effector memory T cells in the liver, lung, and kidney, and somewhat lower frequencies of these cells in the spleen and bone marrow (**Figure 5B**). Upon peptide restimulation of CD8^+^ T cells from the spleen of recipients, we observed similar frequencies of TNF-α^+^IFN-γ^+^ and NUR77^+^ T cells in both CD8^+^ T-cell populations (**Figures 5C, D**).

### TRPM2 Deficiency Does Not Impair CD4^+^ T-Cell Differentiation *In Vivo*

**Figure 6:**
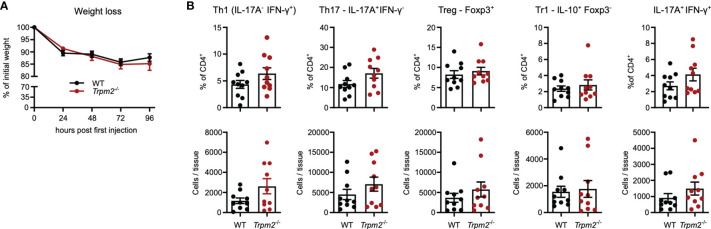
TRPM2 deficiency does not impair the CD4^+^ T-cell response in response to anti-CD3 mAb induced intestinal inflammation. Mice were i.p. injected with anti-CD3 mAb on days 0, 2, and 4 and analyzed 4 h after the last injection. **(A)** Percentage of initial body weight. **(B)** Frequencies and numbers of different CD4^+^ T-cell populations in the small intestinal mucosa. Bars depict mean ± SEM. Results were analyzed with the Mann–Whitney test. Data were pooled from two independent experiments.

In order to test the response of *Trpm2* ^-/-^ CD4^+^ T cells *in vivo*, we used the model of anti-CD3 mAb-induced intestinal inflammation. In this model, repeated injection of anti-CD3 mAb causes systemic T-cell activation. A main hallmark of the model is the activation and accumulation of Th1 and Th17 cells in the small intestine resulting in inflammation of the intestinal mucosa, and diarrhea and weight loss as disease manifestations. As a consequence of the inflammation, Th17 cells differentiate to IL-10-secreting Tr1 cells and enhanced frequencies of both Tr1 cells and conventional Foxp3^+^ Treg cells are found in the small intestinal mucosa. Thus, the anti-CD3 application model allows the analyses of Th1, Th17, and Treg cell responses as well as the formation of Tr1 cells (40–43). Four days after anti-CD3 mAb treatment, mice had lost about 15% of their weight; however, weight loss was similarly extensive in WT and *Trpm2* ^-/-^ mice (**Figure 6A**). Characterization of T cells from the small intestinal mucosa revealed similar frequencies and numbers of CD4^+^ Th1, Th17, Treg, and Tr1 cells in WT and *Trpm2* ^-/-^ mice (**Figure 6B**). Thus, deficiency of TRPM2 did not affect the CD4^+^ T-cell response in this model.

**Figure 7:**
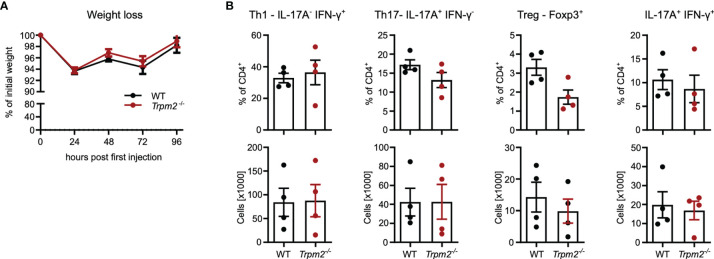
TRPM2 deficiency does not impair response of transferred CD4^+^ T cells in the model of anti-CD3 mAb induced intestinal inflammation. Total CD4^+^ T cells from WT and *Trpm2* ^-/-^ mice were transferred into *Rag1* ^-/-^ mice. After 4 weeks, recipients were treated with anti-CD3 mAb on days 0, 2, and 4 and were analyzed 4 h after the last injection. **(A)** Percentage of initial body weight. **(B)** Frequencies and numbers of different CD4^+^ T-cell populations in the small intestinal mucosa. Bars depict mean ± SEM. Results were analyzed with the Mann–Whitney test.

As discussed in the context of the infection model, TRPM2 deficiency in cells other than T cells could mask an altered CD4^+^ T-cells response. Therefore, *Rag1* ^-/-^ mice were reconstituted with CD4^+^ T cells from either WT or *Trpm2* ^-/-^ mice and then treated with anti-CD3 mAb. Under these conditions, weight loss was less pronounced but similar in mice reconstituted with WT and *Trpm2* ^-/-^ cells (**Figure 7A**). Recipients did not differ in the frequencies and numbers of intestinal IFN-γ^+^ Th1 cells, IL-17A^+^ Th17 cells, and IFN-γ^+^IL-17A^+^ CD4^+^ T cells as well as in frequencies and numbers of Foxp3^+^ cells (**Figure 7B**).

In conclusion, we could not demonstrate a substantial role of TRPM2 in the *in vivo* response of CD4^+^ T cells.

## Discussion

Upon TCR stimulation, we observed a similar induction of IRF4, NUR77, and CD69 after 4, 24, and 48 h in both CD4^+^ and CD8^+^ T cells from WT and *Trpm2* ^-/-^ mice. This result is consistent with the regular early Ca^2+^ signal after TCR stimulation of *Trpm2* ^-/-^ T cells reported by Wolf and colleagues (7) and indicates that TRPM2 is not required for early T-cell activation. In line with this concept, *Trpm2* ^-/-^ CD8^+^ T cells were not impaired in their proliferation after polyclonal stimulation *in vitro* and in their response to *L. monocytogenes* infection *in vivo*. The absence of effect is further consistent with the relatively low expression levels of mRNA for *Trpm2*, *Cd38*, and *Bst1/Cd157* (coding for a NAD-glycohydrolase closely related to CD38 (44) (**Supplementary Figure 1**, Immgen.org (20)) and of TRPM2 protein (**Figures 1A**, **2A** and **Supplementary Figure 2A**) in naive mouse CD4^+^ and CD8^+^ T cells. Our results are in contrast to results from Melzer et al., who reported reduced *in vitro* proliferation and cytokine production of *Trpm2* ^-/-^ T cells (19). Currently, we have no valid explanation for these inconsistent results.

Differentiation of CD4^+^ Th cells is regulated by signals from the environment, in particular inflammatory cytokines, but also by the quality of the TCR-signal (2–4). Thus, TRPM2-facilitated Ca^2+^ signaling could affect Th-cell differentiation. However, under defined *in vitro* conditions, we observed similar Th1, Th17, Tr1, an Treg-cell differentiation of purified WT and *Trpm2* ^-/-^ CD4^+^ T cells. Consistent with the results from the *in vitro* assays, Th-cell differentiation was also only marginally affected in anti-CD3 mAb-induced inflammation, which is associated with intestinal accumulation of Th1, Th17, Tr1, and Treg cells (40–43). Thus, TRPM2 has, at least in our experimental models, no major function in CD4^+^ Th-cell differentiation. Interestingly, *Trpm2* ^-/-^ mice and WT mice developed comparable symptoms to anti-CD3 mAb treatment. This differs to the attenuated disease of *Trpm2* ^-/-^ mice in the dextran sulfate sodium (DSS) model (29). In contrast to the anti-CD3 mAb model in which disease is caused by the response of T cells, the DSS colitis model is primarily driven by innate immune mechanisms after damage and bacterial infiltration of the colon mucosa. Consistent with this notion, the milder course of DSS colitis of *Trpm2* ^-/-^ mice is associated with reduced neutrophil but similar T cell accumulation (29).

TRPM2 could be relevant at later stages of the T-cell response for instance due to increased expression levels of TRPM2 or of ADPR generating enzymes. mRNA expression analysis did not reveal major changes in *Trpm2* levels in different T-cell subsets sorted from naïve and infected mice. In contrast, the expression of CD38 was upregulated on effector and memory T-cell subsets (**Figure 1F** and **Supplementary Figure 1**). However, our results from long-term *in vitro* coculture and the analysis of CD8^+^ T cells 8 weeks post co-transfer and infection argue against such a late function, since we detect no major changes in the ratio of WT and *Trpm2* ^-/-^ donor cells in cell culture and in the spleen and liver, the main organs of *L. monocytogenes* infection in the mouse. Interestingly, there was a slightly reduced accumulation of *Trpm2* ^-/-^ CD8^+^ T cells in the lung and kidney. Thus, TRPM2 might be required for the migration of T cells to these tissues or the survival of T cells within these tissues. The CD38–ADPR–TRPM2 axis has been linked to chemokine signaling in myeloid cells (9), and it is possible that this pathway is also active in chemokine signaling in T cells and thereby required for migration of activated T cells to peripheral tissues such as lung and kidney. However, this function would be restricted to only certain tissues and chemokine receptors since we did not observe impaired migration of CD8^+^ T cells to liver and bone marrow or of CD4^+^ T cells to the intestinal mucosa.

In summary, our results do not support a major function of TRPM2 in T-cell activation and differentiation. However, we cannot exclude a role of TRPM2 in T-cell subsets or differentiation stages not analyzed in our assays. Our analyses were entirely conducted with mouse T cells and in mouse models. Thus, we can also not exclude that TRPM2 has a more substantial role in human T cells.

## Data Availability Statement

The original contributions presented in the study are included in the article/**Supplementary Material**. Further inquiries can be directed to the corresponding author.

## Ethics Statement

The animal study was reviewed and approved by Behörde für Justiz und Verbraucherschutz der Freien und Hansestadt Hamburg.

## Author Contributions

Conceptualization: NL, MN, AHG, SH, H-WM. Data curation: NL, MN, MC, JS, VS. Methodology: NL, MN, TB, SM, FK-N, SH, H-WM. Formal analysis: NL, MN. Project administration: SH, H-WM. Writing, review, editing: all authors. All authors contributed to the article and approved the submitted version.

## Funding

This work was supported in part by the Deutsche Forschungsgemeinschaft (project number 335447717, SFB1328: to SH, H-WM, FK-N, and AHG). SH has an endowed Heisenberg-Professorship awarded by the Deutsche Forschungsgemeinschaft. MN has a scholarship from the Else Kröner Stiftung (iPRIME).

## Conflict of Interest

The authors declare that the research was conducted in the absence of any commercial or financial relationships that could be construed as a potential conflict of interest.

## Publisher’s Note

All claims expressed in this article are solely those of the authors and do not necessarily represent those of their affiliated organizations, or those of the publisher, the editors and the reviewers. Any product that may be evaluated in this article, or claim that may be made by its manufacturer, is not guaranteed or endorsed by the publisher.

## Abbreviations

ADPR, adenosine 5′-diphosphate-ribose; cADPR, cyclic adenosine 5′-diphosphate-ribose; 2dADPR, 2′-deoxyadenosine 5′-diphosphoribose; IP_3_, d-*myo*-inositol 1,4,5-trisphosphate; Lm, *Listeria monocytogenes*; LmOVA, ovalbumin recombinant *Listeria monocytogenes*; NAADP, nicotinic acid adenine dinucleotide phosphate; OVA, ovalbumin; PMA, phorbol 12-myristate 13-acetate; RYR, ryanodine receptor; TPC, two-pore channel; TRPM2, transient receptor potential cation channel subfamily M member 2.
