# Supplementary material for: TRPM2 Is Not Required for T-Cell Activation and Differentiation
Source: Front Immunol. 2022 Jan 14;12:778916. doi: 10.3389/fimmu.2021.778916 (PMC8795911; doi:10.3389/fimmu.2021.778916)
Supplement: Supplementary file 1 [file DataSheet_1.pdf]

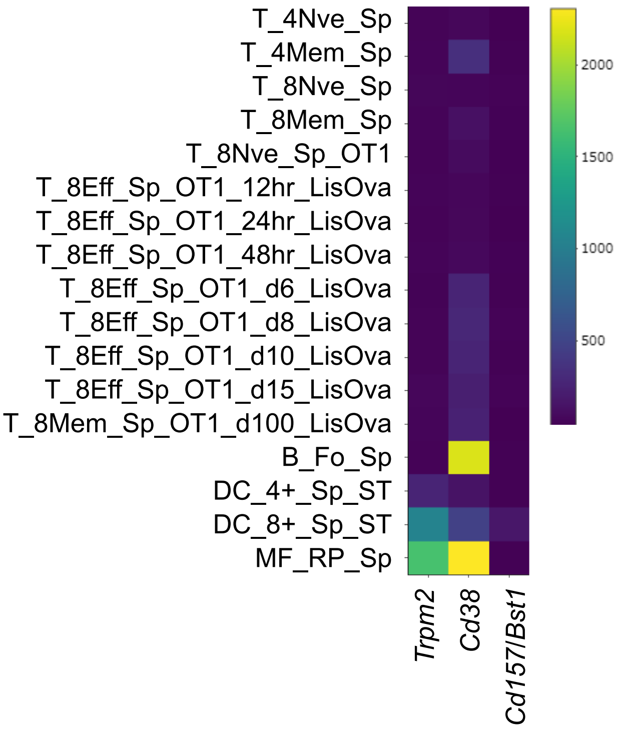

Supplementary Figure 1. mRNA expression of *Trpm2*, *Cd38*, *Bst/Cd157*. mRNA expression for selected mouse leucocyte populations was compiled from the microarray data set of the Immunological Genome Project Consortium (www.immgen.org). MF_RP_Sp: spleen red pulp macrophages; DC_8+_Sp_ST: spleen CD8$^+$ DC; DC_4+_Sp_ST: spleen CD4$^+$ DC; B_Fo_Sp: spleen follicular B cells; T_8Nve_Sp: spleen CD8$^+$ naive T cells; T_8Mem_Sp: spleen CD8$^+$ memory T cells; T_4Nve_Sp: spleen CD4$^+$ naive T cells; T_4Mem_Sp: spleen CD4$^+$ memory T cells; T_8Nve_Sp_OT1: spleen naïve OT-1 CD8 T cells; LisOVA values: OT-1 CD8$^+$ T cells transferred into mice infected with LmOVA and re-isolated at different time point post transfer. Scaling of expression: < 5 traces, 5-20 very low, 20-80 low, 80-800 medium, 800-8000 high.

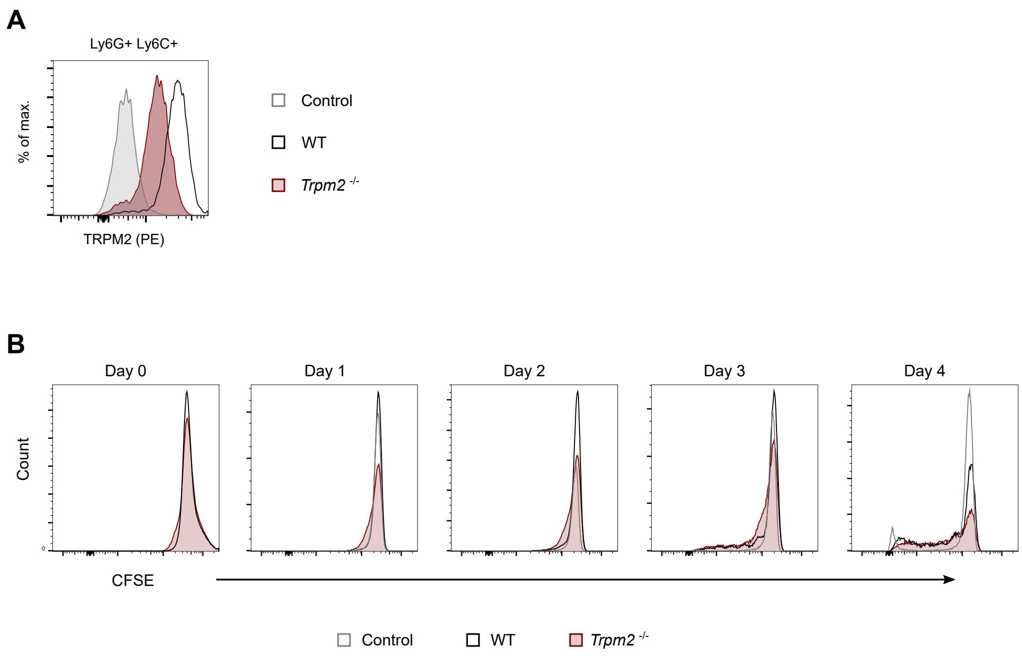

Supplementary Figure 2. TRPM2 expression on myeloid cells and proliferation of CD8$^+$ T cells. (**A**) Ly6C$^+$ and Ly6G$^+$ cells (inflammatory monocytes and neutrophil granulocyte) were intracellularly stained with anti-TRPM2 mAb and PE-conjugated anti-rat IgG antibody (control: WT cells stained only with the secondary antibody). (**B**) Spleen cells were CFSE-labelled and stimulated with anti-CD3ε mAb and anti-CD28 mAb or were cultured without stimulation. Representative results for CFSE intensity on days 0-4 of activated CD8$^+$ T cells from WT and *Trpm2*$^{-/-}$ mice of and non-activated CD8$^+$ T cells from WT mice (control) are shown.

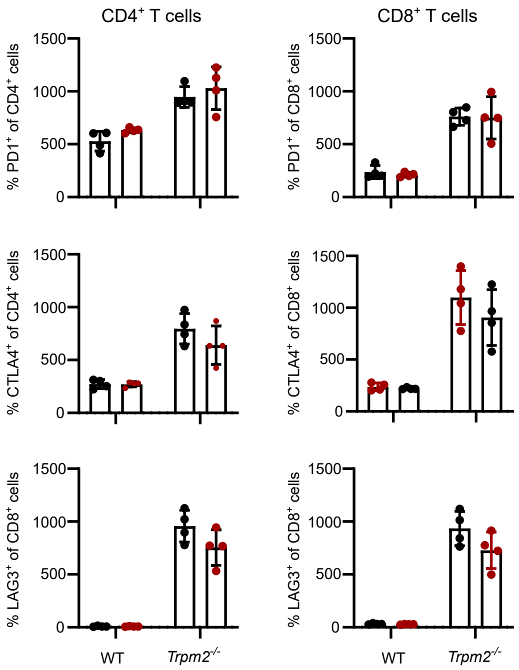

Supplementary Figure 3. Expression of inhibitory receptors on TRPM2-defiecient T cells. Spleen cells from WT and *Trpm2*$^{-/-}$ mice were stimulated for 24h with plate-coated anti-CD3ε mAb and soluble anti-CD28 mAb. Surface expression of PD1 and LAG3 and intracellular expression of CTLA4 was determined by extra and intracellular antibody staining. Bars depict mean ± SEM. Results were analyzed with unpaired t-test.

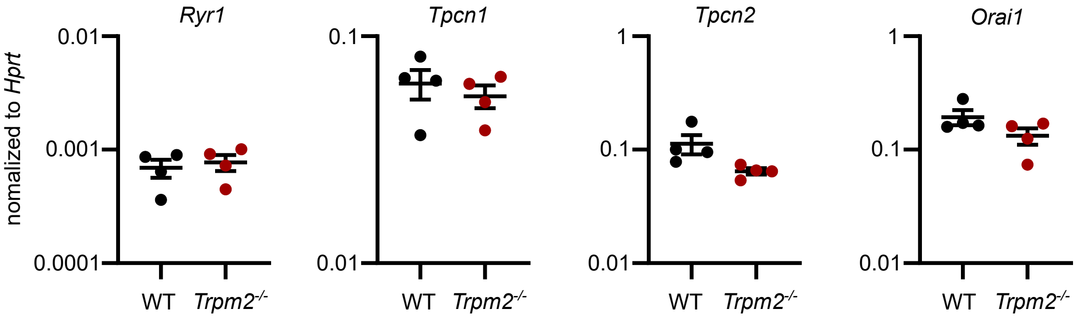

Supplementary Figure 4. Expression of Ca^{2+} channels in *Trpm2*$^{-/-}$ T cells. RNA was isolated from T cells from spleens of WT and *Trpm2*$^{-/-}$ mice. Expression of *Ryr1*, *Tpcn1*, *Tpcn2* and *Orai1* and *Hprt* was quantified in quadruplicates by TaqMan RT-PCR. Expression levels of Ca^{2+} channels were normalized to *Hprt* levels. Results were analyzed with Mann Whitney test.
